# Hyponatremia in the neonatal intensive care unit: incidence, risk factors and effect on mortality

**DOI:** 10.1186/s12887-026-06582-3

**Published:** 2026-02-04

**Authors:** Oğuz Salih Dinçer, Canan Seren

**Affiliations:** 1 Department of Pediatrics, Faculty of Medicine, Ondokuz Mayıs University, Samsun, Turkey; 2 Department of Pediatrics, Division of Neonatology, Faculty of Medicine, Ondokuz Mayıs University, Samsun, Turkey

**Keywords:** Hyponatremia, Newborn, Neonatal intensive care unit (NICU), Incidence, Risk factors, Mortality, Preterm

## Abstract

**Objective:**

The aim of this study was to evaluate the incidence of hyponatremia, risk factors, and its effect on mortality in neonates hospitalized in the neonatal intensive care unit (NICU).

**Methods:**

This retrospective study was conducted in the NICU of a university hospital in Türkiye over a seven-month period in 2016. All 527 infants admitted to the NICU during the study period were included in the study. Hyponatremia was defined as a serum sodium level < 135 mEq/L. Demographic data, clinical diagnoses, fluid therapy, diuretic use, and survival outcomes were analyzed. Multivariate logistic regression analysis was used to identify independent risk factors.

**Results:**

The incidence of hyponatremia in the cohort was 21.2% (112/527). The incidence was higher in preterm (23.6%) and extremely low birth weight (ELBW) infants (61.1%). Hyponatremia was mild in the majority of cases (79.9%). The average gestational age and birth weight of hyponatremic infants were significantly lower than those of non-hyponatremic ones (*p* = 0.011 and *p* = 0.038, respectively). In logistic regression analysis, low gestational age was identified as an independent risk factor for hyponatremia (OR = 0.905, *p* = 0.031). Respiratory system disorders (55.4%) were the most common accompanying comorbidity. Diuretic use in 31.8% and administration of hypotonic fluids (1/5 normal saline) in 45.2% of babies were common associated factors. Mortality was significantly higher in hyponatremic babies when compared with non-hyponatremic ones (10.7% vs. 2.9%; *p* = 0.001).

**Conclusion:**

Hyponatremia is a common and serious condition in the NICU, associated with a significant increase in mortality; with low gestational age being a key independent risk factor. The frequent use of diuretics and hypotonic fluids highlights potential iatrogenic contributions. These findings suggest the need for careful serum sodium monitoring and a re-evaluation of fluid management protocols, especially for vulnerable ELBW preterm infants.

## Introduction

Careful management of fluid and electrolyte balance is essential for delicate homeostasis in the neonatal intensive care unit (NICU). Sodium (Na⁺) plays a key role in regulating body fluid volumes and acid–base balance [1]. Therefore, disturbances in serum Na⁺ concentration may lead to serious clinical consequences, emphasizing the importance of close monitoring and timely intervention to prevent adverse outcomes [2, 3].

In neonates, hyponatremia is defined as a serum Na⁺ concentration below 135 mEq/L (equivalent to mmol/L) [4, 5]. As one of the most common electrolyte disturbances observed in the NICU [6, 7], the reported incidence of hyponatremia ranges from 27% to 52%, with higher rates observed among extremely low birth weight (ELBW) infants [8–10]. Despite its clinical relevance, available data remain heterogeneous across populations and healthcare settings, and comprehensive multicenter studies are scarce, particularly in middle-income regions [7, 11].

Multiple factors may contribute to hyponatremia in newborns, including dehydration, gastrointestinal losses, central nervous system (CNS) pathologies, acute or chronic renal failure, and the syndrome of inappropriate antidiuretic hormone (ADH) secretion [2, 5, 12]. In the neonatal period, hyponatremia represents a significant cause of morbidity and mortality, often occurring in association with complications and comorbidities such as sepsis, seizures, thrombocytopenia, acute kidney injury (AKI), hyperglycemia, and hypocalcemia [3, 7]. Early recognition, effective management, and identification of associated risk factors are essential to prevent adverse outcomes.

The primary objective of this study was to evaluate the incidence and mortality impact of hyponatremia in neonates admitted to NICU. By identifying associated clinical conditions and risk factors, the study aimed to provide evidence to improve prevention and management of hyponatremia in neonatal care.

## Materials and methods

### Study population and data collection

This retrospective study included all infants hospitalized in the NICU of a university hospital in Türkiye between January 1 and July 31, 2016. Cases with at least one serum Na⁺ level < 135 mEq/L constituted the hyponatremia group. Of the 131 infants identified with hyponatremia, 17 babies with pseudohyponatremia (secondary to hyperglycemia) and 2 with laboratory error–related hyponatremia were excluded, leaving 112 infants for analysis.

Patient data were obtained from the hospital information system, NICU records, and patient files. For each case, the following parameters were evaluated: Birth weight, gestational age, gender, primary diagnosis for NICU admission, number of hyponatremic episodes, severity of hyponatremia (mild, moderate, or severe), AKI based on the modified neonatal Kidney Disease: Improving Global Outcomes criteria [13], diuretic use within one week prior to the episode (in the absence of AKI), composition of intravenous (iv) fluids administered before hyponatremia onset, time to correction of hyponatremia, and final outcome (death or discharge).

Serum Na⁺ levels of 130–134.99 mEq/L were classified as mild, 120–129.99 mEq/L as moderate, and < 120 mEq/L as severe hyponatremia [1, 14]. Because infants with prolonged NICU stays could experience multiple hyponatremic episodes, each period in which the serum Na⁺ concentration dropped below 135 mEq/L and subsequently returned to normal was defined as a separate hyponatremic episode.

### Fluid and electrolyte management

The NICU followed a protocol based on the Turkish Neonatal Society (TNS) 2016 guidelines for fluid and electrolyte balance in newborns [15]. Sodium was added to maintenance fluids only after the documentation of adequate urine output. For infants born before 34 weeks of gestation, parenteral nutrition (PN) including amino acids was initiated on the first day of life. Lipids were added the second day. Minimal enteral nutrition was started as soon as the baby was stable, preferably with own mother’s milk. Serum electrolytes were initially measured between 12 and 24 h of postnatal life; subsequently, these were monitored at least twice weekly in clinically stable infants, with more frequent assessments performed as clinically indicated. Extremely low birth weight (ELBW) infants received daily assessments during the first week of life.

Sodium intake was individualized based on serum Na^+^ levels and renal function tests. Data regarding early fluid and nutrition prescriptions (including PN) were abstracted from medical records for each hyponatremic episode. Hyponatremia management was tailored to the serum sodium level, its trend, and the patient’s clinical status. Mild, asymptomatic episodes were managed through close clinical and biochemical monitoring along with a reassessment of fluid composition and sodium intake. Hypertonic saline was reserved for severe, symptomatic hyponatremia presenting with neurological manifestations; however, no such symptomatic episodes occurred in this cohort.

### Statistical analysis

All statistical analyses were conducted using IBM SPSS Statistics for Windows, Version 23.0 (IBM Corp., Armonk, NY, USA). A two-sided p-value of < 0.05 was considered statistically significant. Data distribution was assessed using the Shapiro–Wilk test. Normally distributed continuous variables were expressed as mean ± standard deviation (SD), and non-normally distributed variables as median (range). Categorical variables were presented as frequencies and percentages. Comparisons between hyponatremic and non-hyponatremic groups were performed using the independent-samples t-test for continuous variables and the Chi-square (χ²) or Fisher’s exact test for categorical variables, as appropriate. Multivariate logistic regression analysis was applied to identify independent risk factors for hyponatremia, with the presence of hyponatremia (yes/no) as the dependent variable. Variables significant in univariate analyses or clinically relevant (gestational age, birth weight, sex) were included in the model. Results are reported as odds ratios (OR) with 95% confidence intervals (CI).

### Ethical approval

Ethical approval was obtained from the Ondokuz Mayıs University Ethics Committee (Approval No: B.30.2.ODM.0.02.08/1984; October 26, 2018).

## Results

During the study period, of 527 neonates admitted to the NICU, hyponatremia was identified in 112 cases, corresponding to an overall incidence of 21.2%. Approximately half of the affected infants were preterm (48.2%). The incidence of hyponatremia in preterms was higher than the term babies (23.6% vs. 19.5%, *p* = 0.299), although the difference was not statistically significant. Demographic characteristics of the study population are summarized in Table 1.

**Table 1:** Comparison of baseline characteristics and outcomes between infants with and without hyponatremia

	Hyponatremic (+) (*n* = 112)	Normonatremic (–) (*n* = 415)	*p* value
Gestational age (weeks) (mean ± SD)	35.1 ± 4.4	36.4 ± 3.0	**0.011**
<27 weeks	6 (5.4%)	5 (1.2%)	
27–29⁶⁄₇ weeks	9 (8.0%)	6 (1.4%)	
30–33⁶⁄₇ weeks	16 (14.3%)	59 (14.2%)	
34–36⁶⁄₇ weeks	23 (20.5%)	105 (25.3%)	
37–40 weeks	56 (50.0%)	226 (54.5%)	
>40 weeks	2 (1.8%)	14 (3.4%)	
Birth weight (grams) (mean ± SD)	2419.7 ± 956.4	2719.5 ± 761.6	**0.038**
<1000 g	11 (9.8%)	7 (1.7%)	
1000–1499 g	13 (11.6%)	17 (4.1%)	
1500–2499 g	25 (22.3%)	119 (28.7%)	
2500–4000 g	57 (50.9%)	260 (62.7%)	
>4000 g	2 (1.8%)	12 (2.9%)	
Sex n (%)			0.502
Male	64 (57.1%)	220 (53%)	
Female	48 (42.9%)	195 (47%)	
Preterm Birth n (%)	54 (48.2%)	175(42.2%)	0.150
Birth weight of non-survivors (grams) (mean ± SD)	1769.2 ± 1083.8	1388.3 ± 1029.9	0.476
Gestational age of non-survivors (weeks) (mean ± SD)	31.8 ± 6.8	30.0 ± 4.9	0.564
Mortality	**12 (10.7%)**	**12 (2.9%)**	**0.001**

Comparisons were performed using independent-samples t-test for continuous variables and chi-square test for categorical variables. *p*<0.05 was considered statistically significant. The sum of birth weight categories for the hyponatremia group is 108, as data were unavailable for 4 infants

*SD* standard deviation

179 hyponatremic episodes were recorded among these 112 infants. 85 patients (75.9%) experienced a single episode, occurring within the first two postnatal weeks, while 27 (24.1%) experienced multiple episodes. The median duration for correction of hyponatremia was two days (range:1–15), and no symptomatic episodes were identified (Table 2).

**Table 2:** Characteristics and preceding factors of hyponatremic episodes

Episode Characteristics	
Episodes per patient, median (range)	1 (1–13)
Age at onset (days), median (range)	11 (0–395)
Duration for correction (days), median (range)	2 (1–15)
Symptomatic episodes, n	0
Episode Severity (*n* = 179)	
Mild (130–134.9 mEq/L), n (%)	143 (79.9%)
Moderate (120–129.9 mEq/L), n (%)	28 (15.6%)
Severe (< 120 mEq/L), n (%)	8 (4.5%)
Preceding Factors (*n* = 179)	
Diuretic use within one week prior, n (%)	
None	122 (68.2%)
Furosemide	45 (25.1%)
Spironolactone	12 (6.7%)
IV fluid administration, n (%)	
None	32 (17.9%)
Dextrose only	9 (5%)
1/5 Normal saline (30 mmol/L Na⁺)	80 (44.7%)
Parenteral nutrition (5 mEq/kg/day Na⁺)	33 (18.4%)
Other fluids	25 (14%)

Of the 179 hyponatremic episodes, 74 (41.3%) occurred within the first 7 days of life (early-onset), with a mean onset time of 1.97 ± 1.76 days. At the patient level, early-onset hyponatremia was observed in 69 of 527 infants (13.1%). Among birth-weight subgroups, early-onset hyponatremia was present in 8 of 18 ELBW infants (44.4%) and in 7 of 30 VLBW infants (23.3%).

The majority of episodes were classified as mild (79.9%) or moderate (15.6%), whereas severe hyponatremia was rare (4.5%). Eight severe hyponatremic episodes were observed in 7 patients (serum Na⁺ levels between 109 and 118 mEq/L). These episodes were managed with controlled Na⁺ correction by adjusting maintenance fluids and Na⁺ intake, together with close biochemical monitoring. Notably, no clinically symptomatic episodes (e.g., seizures or overt neurological manifestations) were documented.

Respiratory disorders constituted the most frequent comorbid condition (55.4%), including respiratory distress syndrome (RDS), transient tachypnea of newborn (TTN), and pneumonia, followed by prematurity and congenital heart diseases (CHD) (Table 3). Many infants had multiple concurrent diagnoses, underscoring the multifactorial nature of hyponatremia in this population. No endocrine abnormalities were identified.

**Table 3:** Primary diagnoses of hyponatremic infants

Diagnosis	*n*	%
Respiratory disorders	62	55.4
Respiratory distress syndrome	41	—
Transient tachypnea of the newborn	17	—
Pneumonia	5	—
Prematurity	54	48.2
Cardiac anomalies and rhythm disturbances	46	41.1
Infections	21	18.8
Central nervous system abnormalities	16	14.3
Hypoxic-ischemic encephalopathy / Intracranial hemorrhage	15	13.4
Major congenital anomalies and chromosomal disorders	10	8.9
Blood group incompatibility	8	7.1
Congenital abnormalities of kidney and urinary tract	5	4.5
Acute kidney injury	1	0.9
Other^*^	14	12.5

Patients could have more than one diagnosis

^*^ Niemann–Pick disease, cystic fibrosis, hemophagocytic syndrome, neonatal convulsion

Medication and fluid-related factors also played a notable role. Diuretic exposure preceded 57 episodes involving 36 individual patients. Among these, 20 (55.6%) had CHD and 5 (13.9%) were diagnosed with intracranial hemorrhage. 20 (55.6%) of these patients in this group were born preterm. Among the recorded episodes, the most frequently administered iv solution prior to hyponatremia was one-fifth (1/5) normal saline, containing 0.18% NaCl, ≈ 30 mEq/L Na⁺. It was used in nearly half of the cases before hyponatremia, while PN fluids containing 5 mEq/kg/day of Na⁺ accounted for another substantial proportion (Table 2).

Compared with non-hyponatremic infants, those with hyponatremia had significantly lower gestational age and birth weight (*p* = 0.011 and *p* = 0.038, respectively). The incidence of hyponatremia increased progressively with decreasing birth weight, affecting 50% of very low birth weight (VLBW) and 61.1% of ELBW infants. Logistic regression analysis identified lower gestational age as an independent risk factor for hyponatremia (OR = 0.905, 95% CI: 0.827–0.991; *p* = 0.031), while birth weight and sex were not independently associated.

Overall mortality was markedly higher among infants with hyponatremia compared with those without (10.7% vs. 2.9%; *p* = 0.001). Birth weight and gestational age among non-survivors did not differ significantly between the two groups.

A total of 12 infants with concomitant hyponatremia died during the study period. The median serum Na^+^ level in these cases was 128.5 mEq/L (range: 109–134 mEq/L). Regarding the primary causes of mortality, sepsis was identified in three infants, while complications of extreme prematurity accounted for four deaths. Congenital heart disease was the primary cause in two cases, hemophagocytosis was identified in one infant, and the remaining two infants died due to pulmonary complications.

Among the seven patients with severe hyponatremia, three mortalities occurred during these episodes. The causes of death were attributed to complications of extreme prematurity in one infant and sepsis in another infant with co-existing Down syndrome and CHD. Another patient died during a subsequent mild hyponatremic attack due to pneumonie. Among the survivors, the median time to normalize serum sodium levels was 4 days (range: 2–5 days).

## Discussion

This study confirms that hyponatremia in the NICU patients is not only a frequent finding, with an incidence of 21.2%, but also a condition associated with significant clinical outcomes. This overall incidence observed in our study is near the lower end of the broad range reported in the literature (29.4 to 50%); which may be attributable to the specific characteristics of our population or our unit’s individualized fluid therapy protocols [9, 16, 17]. Nevertheless, the higher rates we observed among premature and VLBW infants are consistent with findings reported by Hao et al. in premature infants [18]. Congruous with the literature, our findings also demonstrated that the vast majority of hyponatremic cases were of mild severity and asymptomatic [17]. All these findings can be explained by the inherent physiological vulnerability of neonates, which stems from factors such as high total body water content, immature renal functions (low glomerular filtration rate and limited tubular sodium reabsorption), and increased ADH secretion [6, 10, 19].

In our study, the incidence of hyponatremia was 50% in VLBW and 61.1% in ELBW infants. Monnikendam et al. reported a 35% incidence of hyponatremia in ELBW infants during the first postnatal week, noting that only 16% remained normonatremic. In our study, similarly, the incidence of early-onset hyponatremia was 13.1% in the whole study population and 44.4% in ELBW babies. The authors emphasized that early-onset hyponatremia is associated not only with increased mortality but also with adverse neurodevelopmental outcomes [20].

Prematurity and low birth weight are among the most potent predictors of hyponatremia [7, 20]. In our cohort, infants with hyponatremia also had significantly lower gestational ages, and multivariate analysis confirmed lower gestational age as an independent risk factor. This finding can be explained by increased natriuresis due to tubular immaturity and insufficient intestinal sodium absorption [6, 18]. Although low birth weight is a known risk factor for hyponatremia, it did not emerge as an independent predictor in our analysis. This finding likely reflects the biological and statistical overlap between gestational age and birth weight, suggesting that gestational age more accurately reflects the renal and intestinal maturity governing sodium balance than body mass alone. Furthermore, the finding that mortality was significantly higher in the hyponatremic group, despite no difference in gestational age or birth weight among the non-survivors in either group, suggests that hyponatremia itself, and/or the clinical predisposing conditions, may directly contribute to mortality. Therefore, careful management of sodium intake and close monitoring of serum Na⁺ levels are crucial, particularly in premature and low-birth-weight infants, to prevent hyponatremia and its associated complications.

Currently, hypo-osmolar fluids with Na⁺ content close to that of human milk are commonly used for iv fluid therapy in NICU patients [21, 22]. However, recommendations made nearly fifty years ago may no longer be applicable under modern NICU conditions and current limits of viability [4, 22]. The latest ESPGHAN guideline recommend initiating Na⁺ and K⁺ supplementation from the first day of life in ELBW and VLBW infants, provided that adequate urine output is confirmed and that high amino acid and energy intake are administered (LoE 2+, RG 0, conditional recommendation) [19]. Studies have suggested that, in preterm infants who fail to demonstrate appropriate postnatal weight loss and have low urinary Na⁺ concentrations during the first postnatal weeks, fluids with higher Na⁺ content may help preserve total body Na⁺ stores and reduce the risk of hyponatremia [4]. The most recent American Academy of Pediatrics guidelines also advise against the use of hypotonic maintenance fluids in children aged 28 days to 18 years; however, they do not provide specific guidance for neonates nor preterm infants [22, 23].

In our study, in 49.7% of hyponatremic episodes, patients had received either 1/5 saline or dextrose-only solutions prior to the onset of hyponatremia. Although the causal relationship between hypotonic fluids and hyponatremia remains debated, recent literature raises concerns that low-Na⁺ iv fluids may increase the risk of hyponatremia in newborns [6, 22, 23]. In the study by Chang et al., including 174 late preterm and term infants, 24% had serum Na⁺ levels < 132 mEq/L and 39% <134 mEq/L at 24 h of life. The infants received 10–12.5% dextrose as maintenance iv fluid, and hyponatremia was particularly pronounced in those who were not yet feeding orally and relied solely on iv fluids. The authors concluded that standard infusion rates and Na⁺-free maintenance solutions likely contributed to hyponatremia and emphasized the need to re-evaluate current fluid management practices for late preterm and term neonates [22]. Similarly, in our cohort, the most frequently administered fluids prior to the onset hyponatremia were 1/5 saline and PN containing 5 mEq/kg/day Na⁺. These findings support the need to reassess the Na⁺ content of iv fluids used in NICUs and advocate for higher Na⁺ supplementation, particularly for VLBW and ELBW babies.

Diuretic use is among the most common medication-related causes of hyponatremia, and its incidence may reach up to 50% in patients receiving these agents [8, 12, 14]. Diuretic-induced hypovolemic hyponatremia typically resolves upon discontinuation of the medication [5, 12]. In a study from Türkiye, the frequency of diuretic use among NICU patients with AKI was 7.1% [24], while another study found a diuretic use rate of 8.5% among neonates [25]. In our study, the rate of diuretic use prior to the onset of hyponatremia, in the absence of AKI, was 31.8%. This observation indicates that diuretics are used relatively frequently in our NICU and underscores the need for greater caution when prescribing these medications.

The pathophysiology of hyponatremia is often complicated by systemic illnesses, in addition to the underlying physiological vulnerabilities. Systemic infections and pulmonary diseases can lead to hyponatremia via increased ADH secretion in response to inflammation or hypoxia [2, 5, 26]. Indeed, our finding that pulmonary disorders were the most frequent accompanying condition in hyponatremic infants strongly supports this relationship. This finding is also consistent with literature reporting that patients with respiratory failure requiring mechanical ventilation have an approximately four-fold increased risk of hyponatremia, and that 13.6% of children hospitalized for respiratory tract infections are hyponatremic [11].

Notably, in our cohort, hyponatremia observed in infants with TTN may have been facilitated by peripartum factors. During the study period, planned cesarean deliveries accounted for approximately 70% of births in our unit, and expectant mothers routinely received only dextrose-containing iv fluids prior to surgery. Peripartum administration of hypotonic fluids has been associated with maternal and neonatal dilutional hyponatremia. In these TTN cases, oxygen requirement was modest (FiO₂ <40%), which is consistent with the typically mild hypoxemia expected in TTN.

This complex etiology of hyponatremia in turn, makes the management complicated. Rapid fluctuations in serum Na⁺ levels during fluid therapy, whether increases or decreases, can precipitate severe CNS complications [2, 6, 27]. In premature and low-birth-weight infants, with limited sodium regulation capacity, a combination of inflammatory (ADH-driven) and iatrogenic (treatment-related) factors can converge to increase both the incidence and severity of hyponatremia. Therefore, careful monitoring of Na^+^ levels and the timely readjustment of iv fluid therapy in this high-risk group are of critical significance to prevent CNS complications and adverse clinical outcomes.

This study has several limitations, mainly related to its retrospective design. Data collection may have been influenced by incomplete or inconsistent medical records, which limits the ability to establish causal relationships. In addition, as the study was conducted in a single tertiary center, the findings may not be fully generalizable to other NICU populations with different patient characteristics or clinical practices. The retrospective design also precluded evaluation of long-term outcomes, such as neurodevelopmental effects, and prevented assessment of temporal changes in the incidence or risk factors of neonatal hyponatremia. Future multicenter prospective studies are needed to validate these findings and to further clarify the clinical implications of hyponatremia in this vulnerable population.

## Conclusion

We demonstrated that the incidence of hyponatremia in the NICU was 21.2%, rising to 61.1% among ELBW infants, with lower gestational age being the major risk factor. Mortality was significantly higher among hyponatremic infants, which indicated that hyponatremia is not merely a biochemical abnormality but also an important marker, influencing clinical outcomes.

The results of the present study challenge the perception of hyponatremia as an inherent condition of preterm birth and emphasize the need to transition from reactive correction to proactive prevention. A careful reassessment of routine iv fluid and diuretic prescription practices is warranted, particularly for vulnerable preterm and low-birth-weight infants. In NICU patients, careful monitoring for hyponatremia, accurate evaluation of its etiopathogenesis, and implementation of individualized management strategies are essential.

## Data Availability

The datasets generated and/or analyzed during the current study are available from the corresponding author on reasonable request.

## Abbreviations

ADH: Antidiuretic hormone
AKI: Acute kidney injury
CI: Confidence interval
CNS: Central nervous system
ELBW: Extremely low birth weight
IV: Intravenous
KDIGO: Kidney Disease: Improving Global Outcomes
LoE: Level of evidence
Na⁺: Sodium ion
NICU: Neonatal intensive care unit
OR: Odds ratio
RG: Recommendation grade
SD: Standard deviation
VLBW: Very low birth weight
